# From the Bottlecap to the Bottleneck: Frequent Esophageal Impaction of Bottlecaps Among Young Males in a Small University Town

**DOI:** 10.1007/s00455-021-10263-x

**Published:** 2021-02-13

**Authors:** Mattis Bertlich, Friedrich Ihler, Jan M. Sommerlath Sohns, Martin Canis, Bernhard G. Weiss

**Affiliations:** 1 Department of Otorhinolarnygology, Head and Neck Surgery, Ludwig-Maximilians University, Marchioninistr. 15, 81377 Munich, Germany; 2 Department of Otorhinolarnygology, Head and Neck Surgery, University Medical Center, Göttingen, Germany; 3 Department of Diagnostic and Interventional Radiology, University Medical Center, Göttingen, Germany; 4 Department of Nuclear Medicine, Center of Radiology, Hannover Medical School, Hannover, Germany

**Keywords:** Fraternity, Foreign body, Esophageal foreign body, Esophageal impaction, Alcohol abuse, Binge drinking

## Abstract

There have been few reports of ingestion of bottlecaps worldwide. However, all of these seemed to be unlikely accidental ingestions with a comic side effect. In contrast to this, the authors of this study found an accumulation of bottlecap ingestions in a small university town. Hence, we conducted a study to investigate the nature of these ingestions. We conducted a retrospective cohort study in a tertiary referral center in a small German university town (Göttingen). All patients that were admitted for esophageal foreign bodies were screened for accidental ingestion of bottlecaps and included in the study at hand. Overall, there were 14 cases of bottlecap ingestion within 12 years. Patients were exclusively male, average age was 23.0 ± 4.2 years, ranging from 18.3 to 35.6 years. In 13 out of 14 cases, association to a fraternity was found. Young men, particularly those belonging to a fraternity, should be beware of bottlecap ingestion when consuming beer in risky rituals in small university towns. Alternatively, competitive beer drinking may generally be avoided.

## Introduction

Constant consumption of alcohol is dangerous to both mental and physical wellbeing. Nonetheless, (regular) consumption of alcohol is deeply embedded into many societies and a frequent, if not obligatory part of social convention. Regular consumption of alcoholic beverages in moderate amounts, in particular of those that are rich in polyphenol, has even been discussed to have beneficial long-term effects on health and wellbeing [1]—even though there are viewpoints that negate any beneficial effect of alcohol consumption [2].

There are many forms of protracted consumption, one of which is binge drinking where ample amounts of alcoholic beverages are ingested in a short period of time with the overall aim of becoming inebriated. When inebriated, the probability of esophageal impaction significantly increases [3].

While the nature of the ingested foreign bodies naturally varies according to age, social surroundings and objects at hand, the authors of this study noted a peculiar cumulation of esophageal impaction of bottlecaps, particularly in context fraternity drinking rituals. Fraternities often cultivate a strong tradition of heavy drinking rituals. While cases of esophageal impaction of bottlecaps in the scientific literature are scarce [4], we felt that this incidentally detected finding was a common clinical picture in a small German university town. Hence, we decided to investigate the nature of these incidents.

## Patients and Methods

The study at hand was registered with the ethics committee of the University of Göttingen under the File No. 29/6/12 An. The need for informed consent was waived since the nature of this study was a purely retrospective analysis; the inhouse standard-of-care was not altered due to this study.

All patients that were admitted to the University Medical Center Göttingen in the period from 01/2004 to 08/2016 and were registered with the Operation and Procedure Classification System code 1-630.1 (rigid esophagoscopy) in combination with the appropriate ICD-10 GM Diagnosis codes were screened for inclusion in this study. The ICD-10 codes screened were

- T17.2—Foreign body in the pharynx.
- T17.3—Foreign body in the larynx.
- T17.4—Foreign body in the trachea.
- T17.5—Foreign body in the bronchus.
- T17.9—Foreign body in the respiratory tract, unspecified.
- T18.0—Foreign body in mouth.
- T18.1—Foreign body in esophagus.
- T18.8—Foreign body in other or multiple parts of alimentary tract.
- T18.9—Foreign body in alimentary tract, part unspecified.

Moreover, cases were detected by a full text search within the radiology findings database using the German term for bottlecap (“Kronkorken”). The University Medical Center Göttingen is a tertiary referral center and an academic teaching hospital of Göttingen University.

Patients that had claimed to have ingested a bottlecap during their anamnesis or where the diagnosis of ingestion of a bottlecap was based on an incidentally detected finding by radiography or endoscopy were included.

Patient files were used to collect data about the date of ingestion, the patients age at ingestion, the radiologic documentation, bloodwork, the general and specific anamnesis of the patient, the symptoms with which the patient presented, the localization of the bottlecap as well as the treatment and the follow-up of the patients.

Patients were routinely treated either by the Otorhinolaryngology or the Gastroenterology Department; the individual reports from the interventions were then analyzed for the study as well. By otorhinolaryngologists a rigid endoscopy in general anesthesia was performed; a flexible endoscopy was performed by the gastroenterologists in sedation.

Fraternity association was deduced from the patients addresses of residency or where the incident took place and comparison of these addresses with that of local fraternities. Additionally, available membership lists the *Kösener Corpslisten* and the *Mitgliederverzeichnis des Cartellverbandes* were used to cross-reference fraternity association.

## Results

Overall, 14 patients presented with an ingested bottle cap between 01/2005 and 10/2014 were included in the study. The study population was exclusively male with an average (physical) age of 23.0 ± 4.2 years at the time of treatment with the eldest patient being 35.6 years and the youngest 18.3 years. Fraternity association was confirmed in 13 patients.

Upon first presentation, 12 patients reported a definitive ingestion of a bottlecap; in two patients there was no information available as to why the patients were admitted to the ER. All patients presented within 24 h after ingestion. Symptoms described were in all patients odyno-, dys- or aphagia, foreign body sensation and/or retrosternal pain. No patient was actually capable of properly swallowing. In nine patients, there was a positive anamnesis for alcohol consumption; in the other five cases, there were no information on previous alcohol consumption available in the patient files. Average blood alcohol concentration was 1.2 ± 0.8 g/l (Table 1).

**Table 1:** Patient and treatment characteristics

		Data available [number (percent)]	
Age [years]	23 ± 4.2	14 (100%)	
Sex [males]	14 (100%)	14 (100%)	
Nobility	5 (35.8%)	14 (100%)	
Fraternity Association	13 (92.3%)	14 (100%)	
Anamnesis for alcohol consumption	9 (100%)	9 (64.3%)	
Blood alcohol concentration [g/l]	1.2 ± 0.8	7 (50.0%)	
Anamnesis for bottlecap ingestion	12 (100%)		12 (85.7%)
Bottlecap localized radiologically	14 (100%)		14 (100%)
Rigid endoscopy	7 (50%)		14 (100%)
Flexible endoscopy	7 (50%)		14 (100%)
Mucosal lesion	10 (76.9%)		13 (92.9%)
Successful recovery of bottlecap	14 (100%)		14 (100%)

**Fig. 1:**
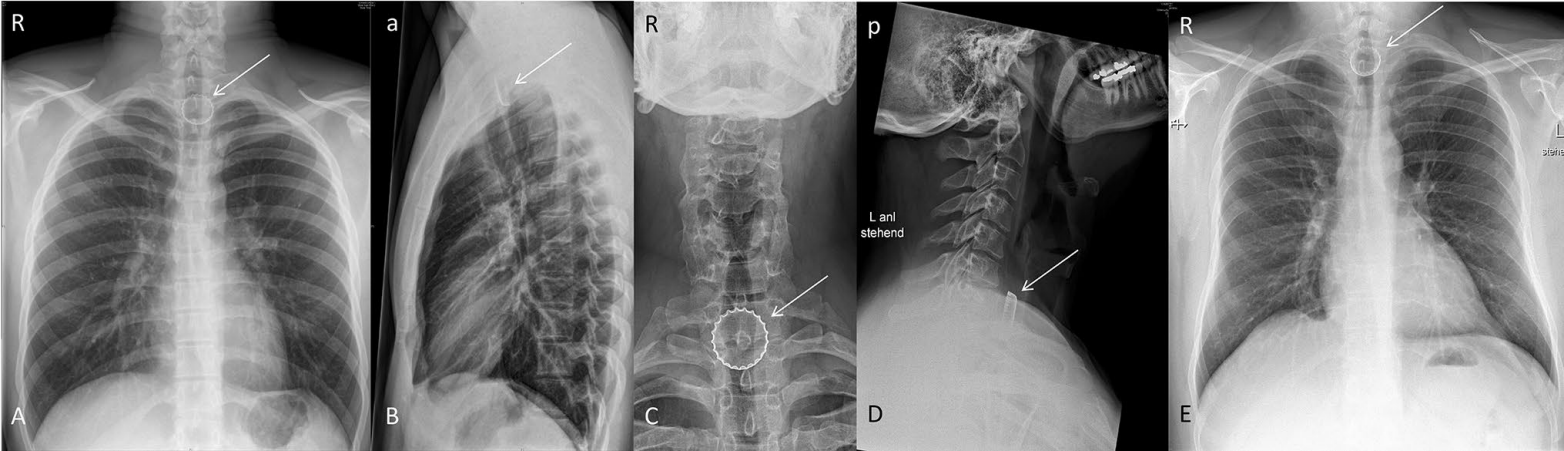
Upper esophageal narrowing. The upper esophageal narrowing lies at the level of near cervical vertebrae VI/VII, about 15 cm measured from the front row of teeth. **A**, **B**: Posterior–anterior X-ray (**A**) and left-sided lateral X-ray of the thorax (**B**). Bottlecap near thoracic vertebra I/II (arrow). **C**, **D**, **E**: Anterior–posterior (**C**) and left-sided lateral X-ray of the cervical spine (**D**). Posterior–anterior X-ray of the thorax (**C**). Bottlecap near cervical vertebra VII and thoracic vertebra I (arrow). *R* right; *L* Left; *a* anterior; *p* posterior

**Fig. 2:**
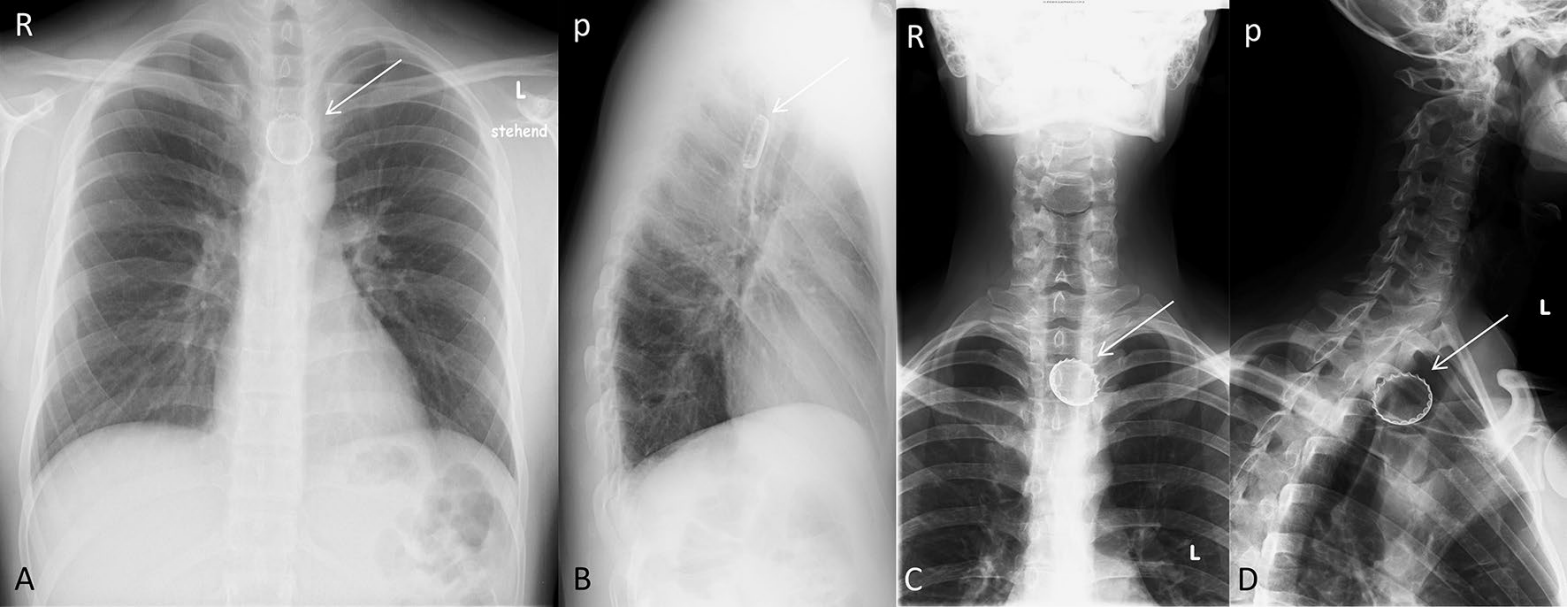
Middle esophageal narrowing. Point where the esophagus passes by the aorta and the left main bronchus. It lies at the level near of thoracic vertebrae IV. This is about 10 cm caudal to the upper narrowness. **A**, **B**: Posterior–anterior X-ray of the thorax (**A**) and left-sided lateral X-ray. Bottlecap near thoracic vertebra IV/V (arrow). **C**, **D**: Anterior–posterior (**C**) and left-sided lateral X-ray of the cervical spine (**D**). Bottlecap near thoracic vertebra II/III (arrow). *R* right; *L* Left; *a* anterior; *p* posterior

**Fig. 3:**
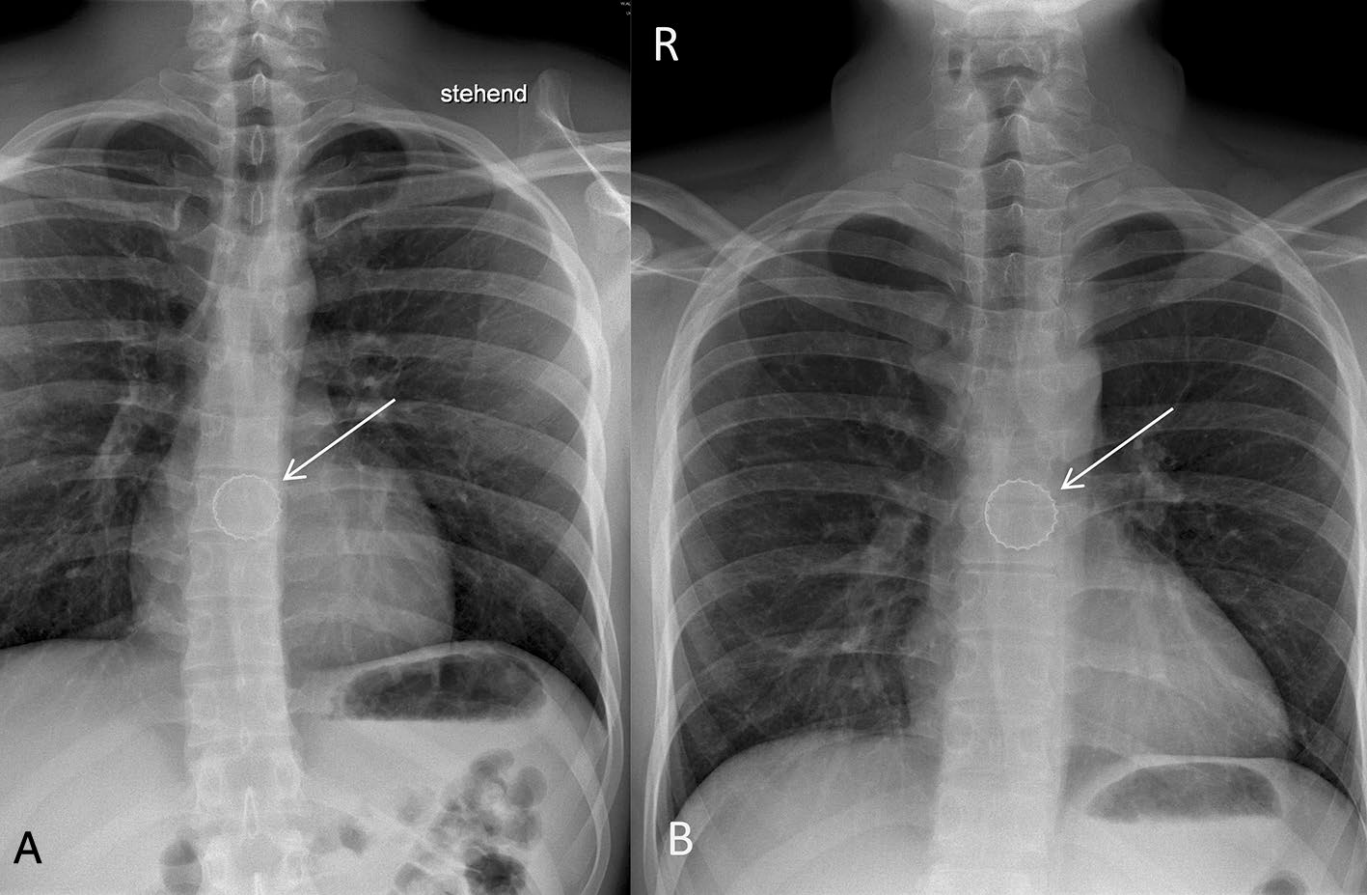
Lower esophageal narrowing. It lies at thoracic vertebrae near X/XI in the area of the esophageal hiatus shortly before the esophago-gastral transition. **A**: Posterior–anterior X-ray of the thorax (**A**). Bottlecap near thoracic vertebra VII (arrow). **B**: Posterior–anterior X-ray of the thorax (**A**). Bottlecap near thoracic vertebra VI/VII (arrow). *R* right; *L* Left; *a* anterior; *p* posterior

In all patients, there was radiographic proof by chest or neck X-ray of a bottlecap stuck in the esophagus. 10 (71.4%) were localized in the cranial third of the esophagus, 3 (21.4%) were localized in the middle third of the esophagus and 1 (7.1%) was localized in the caudal third of the esophagus (Figs. 1, 2, 3). Interestingly, in all imaging findings, the bottlecap has nearly the same alignment in space, flat parallel to the spine.

7 (50%) of the patients were treated by the Department of Otorhinolaryngology and 7 (50%) were treated by the Gastroenterology Department. In all of the patients treated by rigid esophagoscopy, the bottlecap was successfully recovered upon the first attempt (7 of 7, 100%). It was removed by using an appropriate grasping forceps. In three of the seven patients treated by flexible endoscopy, the bottlecap was accidentally lodged into the stomach and subsequently removed by endoscopy within the same procedure in one case. Two of those cases (2 of 7, 28.6%) required a second day procedure. During flexible endoscopy the bottlecap was removed by appropriate grasping forceps in 5 cases, and using a mesh loop in 2 cases. In two cases, a “golden bottlecap” was reported or documented. 76.9% showed mucosal lesions after removal of the bottlecap. A control endoscopy after successful removal was performed in two of those cases. A nasogastric feeding tube has not been placed in any case.

All patients were discharged the same day or within the following 48 h after bottlecap removal. None of the patients were readmitted for the same or another bottlecap. In the further follow-up, a 22-year-old patient died 18 months after removal of the bottlecap in an alcohol-related incident.

## Discussion

Firstly, we found in the cohort at hand that the ingestion of bottlecaps seems to be—albeit the foreign body is relatively sharp and its ingestion regularly associated with intoxication—not particularly dangerous: There was no sign of advanced esophageal damage apart from local erythema or slight mucosal erosions. This is probably owed by the fact that the diagnostic and clinical challenges after bottlecap ingestion are not particularly difficult: The majority of patients actually state to have previously ingested a bottlecap, and diagnosis is safely confirmed by X-ray of the neck or thorax. Moreover, all patients presented shortly after the incident. However, this does not mean bottlecap ingestion into the esophagus is not a serious issue—it is our conviction that the relatively harmless numbers are mostly due to the small sample size and the early admission to the hospital. In addition to this, the collective is young and—apart from the underlying intoxication—healthy, making a serious course of the condition less likely. Finally, these cases highlight an important aspect when it comes to foreign bodies that are strange in nature: every account needs to be taken seriously.

Consequently, the subsequent removal is easily feasible either by rigid or flexible endoscopy and can regularly be achieved without further complications, as recommended by the appropriate guidelines [3, 5]. Generally speaking, flexible endoscopy for removal of foreign bodies may be associated with a little less discomfort and hence favorable [5, 6]. However, as far as conclusions may be drawn from the data at hand, in terms of bottlecaps rigid endoscopy may be favorable over flexible endoscopy. This is since the latter lodged the bottlecap into the stomach in several cases and eventually even required additional procedures. Moreover, rigid endoscopy may provide additional protection when retracting the foreign body.

However, what is striking about the collective at hand is not so much the conclusion that may be drawn from the handling of the esophageal foreign bodies. There is an ample amount of scientific literature addressing esophageal foreign bodies [7, 8], including numerous reports of remarkable foreign bodies [9, 10] and even scientific guidelines [3]. What is remarkable about the collective at hand is both the homogeneity of the foreign bodies as well as the patients characteristics: It has been established that bottlecaps pose a considerable danger to the eyes [11] the knee [12] and, if severely misplaced, even the urogenital tract [13]. Hence one might deduce that bottlecaps themselves are accidents just waiting to happen, and that beer and other soft drinks should henceforth only be sold in soft cartridges like milk. However, the fact that there were 14 cases of bottlecap ingestion within a 10-year period in a city of only approximately 100,000 inhabitants needs to be put in perspective: In scientific literature, we found six cases of ingestion of bottlecaps in a 30-year period worldwide, with reports coming from Australia, the USA, Belgium, Poland and Germany, and a considerable heterogeneity on age (yet not gender) and how the bottlecap was ingested. (Table 2) [4, 14–18] The collective at hand on the other hand is quite homogenous. However, the great common denominator of both the aforementioned reports as well as the case series at hand is the gender of the subjects. The subjects were exclusively male. While there are numerous potential explanations for this observation, we believe that this observation goes hand in hand with another aspect of the collective at hand: the association to fraternities.

**Table 2:** Worldwide case reports of bottlecap ingestion

Authors	Journal	Year	Country	Subject sex and age
Rottmann SJ et al.	Ann Emerg Med	1988	USA	M/19
Prakash K et al.	N Engl J Med	1989	USA	M/36
Douglas RJ	BMJ	2007	Australia	M/24
Debonnaire P et al.	Eur Heart J	2013	Belgium	M/38
Rojek L et al.	Endoscopy	2017	Poland	M/22
Werner CR et al.	Gastroenterology	2017	Germany	M/22

It is our conviction that the ingestions of said bottlecaps occurred during the academic drinking rituals which have a long, ongoing and strong tradition amongst German fraternities. The American author Mark Twain used to describe these occasions in Heidelberg, Germany in his report “A Tramp Abroad”: “Nine-tenths of the Heidelberg students wore no badge or uniform; the other tenth wore caps of various colors, and belonged to social organizations called “corps.” (…) The “KNEIP” seems to be a specialty of theirs, too. Kneips are held, now and then, to celebrate great occasions, (…). The solemnity is simple; the five corps assemble at night, and at a signal they all fall loading themselves with beer, out of pint-mugs, as fast as possible, (…) the one who has drank the greatest number of pints is proclaimed king. I was told that the last beer king elected by the corps—or by his own capabilities—emptied his mug 75 times. No stomach could hold all that quantity at one time, of course—but there are ways of frequently creating a vacuum, which those who have been much at sea will understand” [19]. While the times and occasions obviously have changed over the course of time, the general idea of (binge) drinking in fraternities is very much the same to this day. Members of different fraternities meet in gloomy basements, and disproportionate quantities of beer are consumed from large vessels over short periods of time as a competition between the fraternities. Numerous beer bottles are opened hastily with anything sharp-edged, flinging the bottlecaps elliptically into the void—the probability of one ending up in a drinking vessel is not a matter of probability, but of time.

This kind of alcohol rich lifestyle is still practiced, promoted and even commercially marketed to young fraternity students in Germany, as is vividly documented in public accounts in social media like *Octopott* or *Korpo-Meme*. Hence, it is very likely that these bottlecaps were accidentally ingested when large quantities of alcohol were hastily consumed as part of fraternity rituals—very much like institutionalized binge drinking.

So how to prevent bottlecap ingestion in fraternity students? It has been suggested that champagne is a significantly safer choice as a beverage [4], since the ingestion of a champagne cork is anatomically significantly more difficult and has—to the best of our knowledge—not yet been reported in scientific literature. However, conservative institutions like fraternities are not terribly prone to change, and regular consumption of champagne might pose a pecuniary challenge for university students. So after careful and lengthy debate amongst the authors of this paper, we recommend the change of beer brand as a first step in increasing the safety of fraternity students. A beer that may be more appealing to the palate rather than the pocket is possibly drunk in a slower habit, and thus the risk on hastily bottlecap ingestion is reduced. Moreover, we believe that serving cold beer could also decrease the risk of bottlecap ingestion—while at the same time being more enjoyable. Fast ingestion of cold food or beverages is known to cause ice cream induced headaches (or colloquially “brain-freeze”) [20]. The attempt of drinking a large amount of ice-cold beer in a short period of time would most likely be unpleasant to the drinker (and rather entertaining to bystanders).

However, all these (comic) considerations aside, the fact that fraternity rituals regularly caused bottlecap ingestions is considerable cause for concern: Any ingestions of a foreign body is harmful and potentially lethal [21], particularly when inebriated. Moreover, fraternity rituals have repeatedly been reported to be harmful and even lethal [22]. Hence it is not surprising that one patient that was included in the study at hand eventually died in an alcohol-related incident within a short period of time. While the authors of this study do not wish to demonize neither fraternities nor their rituals, the dataset at hand should at least raise concern over some of the respective drinking habits.

## Conclusion

We found a remarkably homogenous collective of young men that had previously ingested bottlecaps. All patients presented with a clear anamnesis of bottlecap ingestion, and diagnosis was safely confirmed by X-ray. Removal was easily feasible by either flexible or rigid endoscopy; however, the authors would recommend rigid over flexible endoscopy. We believe bottlecap ingestion was due to fraternity drinking rituals. While all patients easily recovered after surgical bottlecap removal, one patient eventually died in an alcohol-related incident. This highlights the considerable dangers of excessive drinking rituals, as they are—to this very day—practiced in fraternities, drinking societies and likewise.

## Data Availability

The authors of this manuscript declare that the data at hand have not been published, submitted or used in any other manuscript elsewhere. The original, anonymous dataset is available upon request from the corresponding author.

## References

[1] Yuan H, Marmorstein R (2013). Biochemistry. Red wine, toast of the town (again). Science.

[2] Burton R, Sheron N (2018). No level of alcohol consumption improves health. Lancet (London, England).

[3] Chirica M, Kelly MD, Siboni S, Aiolfi A, Riva CG, Asti E (2019). Esophageal emergencies: WSES guidelines. World J EmergSurg.

[4] Douglas RJ (2007). Champagne: the safer choice for celebrations. BMJ.

[5] Gmeiner D, von Rahden BHA, Meco C, Hutter J, Oberascher G, Stein HJ (2007). Flexible versus rigid endoscopy for treatment of foreign body impaction in the esophagus. SurgEndosc.

[6] Popel J, El-Hakim H, El-Matary W (2011). Esophageal foreign body extraction in children: flexible versus rigid endoscopy. SurgEndosc.

[7] Zhong Q, Jiang R, Zheng X, Xu G, Fan X, Xu Y (2017). Esophageal foreign body ingestion in adults on weekdays and holidays: a retrospective study of 1058 patients. Medicine (Baltimore).

[8] Shih C-W, Hao C-Y, Wang Y-J, Hao S-P (2015). A new trend in the management of esophageal foreign body: transnasalesophagoscopy. Otolaryngol neck SurgOff J Am AcadOtolaryngol Neck Surg.

[9] Wuestenberghs F, Druez P (2016). Unusual esophageal foreign body. Gastroenterology.

[10] Vernekar R, Setia MS (2018). Presentation of an unusual metallic foreign body in a child. J Family Med Prim Care.

[11] Cavallini GM, Martini A, Campi L, Forlini M (2009). Bottle cork and cap injury to the eye: a review of 34 cases. Graefes Arch ClinExpOphthalmol.

[12] Boyle S, Talbot JC, Bismil Q, Schilders E (2010). Arthroscopic removal of a plastic soft drink bottle cap in the knee: a case report. Cases J.

[13] Evans JM, South MM, Karram MM (2012). Vesicovaginal fistula due to remote history of vaginal foreign body. Female Pelvic Med ReconstrSurg.

[14] Debonnaire P, De Backer O, Orlent H, Vandevelde K (2013). One for the road. Eur Heart J.

[15] Prakash K, Rosario PG, Kim S (1989). Esophageal obstruction from a beer-bottle cap. N Engl J Med. United States.

[16] Rottman SJ, Lindsay KL, Kuritzkes R (1988). Of college fads, bottle caps, and esophageal obstruction. Ann Emerg Med. United States.

[17] Rojek L, Jasinski A, Adrych K (2017). Endoscopic removal of metal bottle cap from the esophagus. Endoscopy.

[18] Werner CR, Kramer U, Goetz M (2017). Undesired final of a student’s Beer drinking duel. Gastroenterology.

[19] Twain M. A Tramp Abroad. 1880; Chapter IV, accessed online. Available from: https://www.gutenberg.org/files/119/119-h/119-h.htm#ch4.

[20] Kaczorowski M, Kaczorowski J (2002). Ice cream evoked headaches (ICE-H) study: randomised trial of accelerated versus cautious ice cream eating regimen. BMJ.

[21] Parikh MP, Garg R, Gupta N, Sarvepalli S, Singhal A, Lopez R (2020). National trends in healthcare outcomes and utilization of endoscopic and surgical interventions in patients hospitalized with esophageal foreign body and food impaction. Dis esophagus Off J IntSoc Dis Esophagus..

[22] Boglioli LR, Taff ML (1995). Death by fraternity hazing. Am J Forensic Med Pathol.

